# Amyloid β-Peptide Effects on Glucose Regulation Are Dependent on Apolipoprotein E Genotype

**DOI:** 10.1523/ENEURO.0376-22.2023

**Published:** 2023-04-25

**Authors:** Jin Hee Sung, Yang Ou, Steven W. Barger

**Affiliations:** 1 Department of Geriatrics, University of Arkansas for Medical Sciences, Little Rock, AR 72205; 2 Department of Neurobiology and Developmental Sciences, University of Arkansas for Medical Sciences, Little Rock, AR 72205; 3 Geriatric Research, Education and Clinical Center, Central Arkansas Veterans Healthcare System, Little Rock, AR 72205

**Keywords:** Alzheimer’s disease, amyloid β-peptide, apolipoprotein E, glucose, insulin, microtubule-associated protein Tau

## Abstract

The apolipoprotein E gene (*APOE*) confers the greatest genetic risk factor for Alzheimer’s disease (AD), wherein the ε4 allele confers an elevated risk compared with the ε3 allele. Biological mechanisms that differ across these alleles have been explored in mouse models wherein the murine *Apoe* gene has undergone targeted replacement with sequences encoding human ApoE3 or ApoE4 (ApoE-TR mice). Such models have indicated that the two variants of ApoE produce differential effects on energy metabolism, including metabolic syndrome. However, glucose regulation has not been compared in ApoE-TR mice with and without amyloid β-peptide (Aβ) accumulation. We crossed ApoE3-TR and ApoE4-TR mice with a transgenic line that accumulates human Aβ_1-42_. In male ApoE3-TR mice, introduction of Aβ caused aberrations in glucose tolerance and in membrane translocation of astrocytic glucose transporter 1 (GLUT1). Phosphorylation of Tau at AD-relevant sites was correlated with glucose intolerance. These effects appeared independent of insulin dysregulation and were not observed in females. In ApoE4-TR mice, the addition of Aβ had no significant effects because of a trend toward perturbation of the baseline values.

## Significance Statement

Metabolic changes may have a larger interaction with Alzheimer’s disease (AD) pathology and its consequences in individuals who do not carry an *APOE* ε4 allele. The fact that ApoE4 generally failed to exacerbate the effects of amyloid β (Aβ) on glucose further highlights the growing distinction between the glycemic effects of Aβ versus those of peripheral insulin resistance.

## Introduction

Alzheimer’s disease (AD) is a chronic and irreversible brain disorder that involves memory loss and decline of cognitive ability. Risk for developing AD is elevated in a dose-dependent manner by the ε4 allele of the gene (*APOE*) for apolipoprotein E (ApoE), relative to the more common ε3 allele. A wealth of genetic, biochemical, and neuropathological evidence indicates that CNS accumulation of amyloid β-peptide (Aβ)—either as plaques or in a more diffuse, even soluble, form—is the precipitating etiologic event for AD. Accumulation of Aβ is higher in individuals with an *APOE* ε4 allele. This relationship has led to the hypothesis that *APOE* ε4 is associated with higher rates of AD because its protein product (ApoE4) promotes Aβ aggregation or retention in the brain. However, other mechanisms are possible, and considerable evidence indicates that the protein product of the *APOE* ε4 allele (ApoE4) enters the cell nucleus and binds specific gene enhancer elements (Parcon et al., 2018; Lima et al., 2020).

One of the earliest events in AD pathogenesis after CNS accumulation of Aβ is a decline in cerebral metabolism of glucose (CMR_glc_; Jack et al., 2000). The decline in CMR_glc_ is highly correlated with cognitive impairment, and it has impressive specificity as a predictor of progression from mild cognitive impairment (MCI) to frank dementia (Smailagic et al., 2018; Shen et al., 2019) or from normal cognition to MCI (Reiman et al., 1998; de Leon et al., 2001; Mosconi et al., 2008). We recently determined that CMR_glc_ contributes to somatic glucose tolerance, as a transgenic mouse line that accumulates Aβ in the CNS has both reduced CMR_glc_ and impaired glucose tolerance. This appears to result from a decrease in the fraction of glucose transporter 1 (GLUT1) subcellularly localized to the plasma membrane of astrocytes (or other CNS parenchymal cells; Hendrix et al., 2021). This effect on the 45-kDa form of GLUT1 was also observed in human cerebrum and was the only significant alteration in glucose transporters we identified; reduction of GLUT3, the only CNS transporter that is unique to neurons, does not suppress CMR_glc_ (Stuart et al., 2011).

Persons homozygous for the ε4 allele of *APOE* show an age-related drop in CMR_glc_ in the absence of—or, implicitly, before—cognitive impairment (Small et al., 1995; Reiman et al., 1996, 1998). Female mice carrying targeted replacement (TR) of the murine *Apoe* gene with sequences encoding human ApoE4 show impaired glucose tolerance compared with ApoE3-TR mice (Koren-Iton et al., 2020), but this difference may disappear when compounded by the pathologies associated with diet-induced obesity or Aβ accumulation (Christensen and Pike, 2019; Koren-Iton et al., 2020). The latter manipulation was performed through transgenesis for the full amyloid precursor protein (APP), which has marked effects on peripheral glucose regulation (Needham et al., 2008; Tu et al., 2012; Botteri et al., 2018; Kulas et al., 2019).

Here, we sought to determine the impact of human ApoE4 on glucose regulation in a mouse model that suffers CNS Aβ accumulation without an APP transgene. Unlike prior studies, we directly compared ApoE-TR mice with and without human Aβ expression. To gain greater understanding of the point(s) in brain glucose metabolism impacted by *APOE* genotype, we performed glucose tolerance tests (GTTs) and insulin tolerance tests, and we looked for relationships between these parameters and markers of AD-related pathology, including reduced membrane trafficking of GLUT1, Tau hyperphosphorylation, and interleukin-1β (IL-1β) expression. Glucose uptake rates in primary astrocytes derived from ApoE-TR mice was also assayed to scrutinize events happening at the cell-autonomous level.

## Materials and Methods

### ApoE-targeted replacement mice

Mice homozygous for ApoE3-TR or ApoE4-TR loci (Sullivan et al., 1997) were assessed alongside ApoE-TR mice from the same breeding colony that were also carrying an Aβ_1-42_ transgene (Aβ-Tg) hemizygously, the latter generated by crossing the ApoE-TR lines with the BRI-Aβ42 line. These “Aβ-Tg” mice achieve high expression and release of human Aβ_1–42_ through transgenesis with a fusion protein construct that is transported to the plasmalemma, where the Aβ portion is released by furin protease (McGowan et al., 2005); this model thus avoids APP overexpression, which could otherwise confound glucose regulation (Kulas et al., 2019). Mice were maintained on a 12/12 h light/dark cycle (ZT0 = 6 A.M.) at 23°C with free access to food and water. Both males and females were used. All animal experiments were conducted according to the Institutional Animal Care and Use Committee of the Central Arkansas Veterans Healthcare System (CAVHS), which is certified by the Association for Assessment and Accreditation of Laboratory Animal Care International (AAALAC). Mice were anesthetized in a CO_2_ chamber. Left brains were collected, snap-frozen in liquid N_2_, and stored at −80°C for biochemical assays.

### Genotyping

Blood samples from tail nicks were collected on Whatman FTA card (Millipore Sigma). Total genomic DNA from blood was isolated using REDExtract-N-Amp Blood PCR kit (Millipore Sigma) following the manufacturer’s instructions. PCR was performed on the extracted DNA with the following primers: BRI-Aβ42 forward: 5'-AAG GCT GGA ACC TAT TTG CC- 3′, reverse: 5'-TAG TGG ATC CCT ACG CTA TG-3′; APOE-TR forward: 5'-GGT AGC TAG CCT GGA CGG-3′, reverse: 5'-GCT AGA ACC AGC AGA GAC CC-3′; *Apoe*-WT forward: 5'-GTC TCG GCT CTG AAC TAC TAT-3′, reverse: 5'-GCA AGA GGT GAT GGT ACT CG-3′.

### GTT and ITT

Glucose tolerance test (GTT) was performed at seven weeks or 12 months of age. Mice fasted for 6 h, and then D-(+)-glucose (2 g/kg) was injected intraperitoneally. Concentration of glucose in tail-vein blood ([Glc]_b_] was assayed before injection and at 15, 30, 60, and 120 min after injection using an AlphaTRAK Glucose Meter (Zoetis Inc). Area under the curve (AUC) was calculated as total AUC (tAUC), which compares absolute values of [Glc]_b_. Insulin tolerance test (ITT) performed to assess insulin sensitivity at seven weeks of age. Nonfasted mice were transferred to individual cages injected with 1 U/kg insulin (Humulin R, Lily) intraperitoneally. Blood glucose was measured by the same method as for GTT. Because ITT produces a downward deflection from baseline, integration of these values was performed as area over the curve (AOC), measured from the initial [Glc]_b_.

### Western blot analysis and Wes immunoassay

Harvested brain tissue was stored at −80°C and then chilled further in liquid N_2_ before being pulverized with a liquid N_2_-chilled mortar and pestle. For conventional Western blot analysis, pulverized tissue was combined with RIPA buffer (50 mm Tris-HCl, 150 mm NaCl, 1% NP-40, 0.5% sodium deoxycholate, 0.1% SDS, 1 mm Na_3_VO_4_, 1 mm NaF) containing 5 mg/ml protease inhibitors (Pierce Mini Tablets, EDTA-free), pH 7.5) and sonicated on ice. The lysates were quantified for protein with the Pierce BCA protein assay kit (Thermo Fisher Scientific). Total protein (30 μg per lane) was separated by SDS-PAGE using 4–15% Tris‐glycine gels (Bio‐Rad Laboratories) and transferred to PVDF membrane (Millipore Sigma). The blots were blocked with SuperBlock Blocking Buffer (Thermo Fisher Scientific) for 1 h at room temperature and then incubated with primary antibody overnight at 4°C: AT8, detecting phosphorylated Tau (Thermo Fisher Scientific, MN1020, 1:250), a phospho-independent anti-Tau antibody (Cell Signaling, #46687, 1:1000), or an anti-IL1β antibody (ABclonal, #A16228, 1:1000). The membranes were washed in Tris-buffered saline containing 0.1% Tween 20 (TBST) and then exposed to secondary antibody: anti-rabbit and anti-mouse HRP conjugated (Thermo Fisher Scientific). After washing in TBST, the membranes were reacted with enhanced chemiluminescent substrate (Thermo Fisher Scientific). Protein bands were visualized by exposure to x-ray film and quantification performed with ImageJ.

For analysis of GLUT1, membrane fractionation was performed with a Minute Plasma Membrane Protein Isolation and Cell Fractionation kit (Invent Biotechnologies), which allows separation of tissue homogenates into plasma membrane (PM), cytosolic, and organellar fractions. As previously described (Hendrix et al., 2021), these fractions were analyzed on a SimpleWes capillary electrophoresis device (Protein Simple), which provides requisite separation of the molecular-weight moieties and quantitation. The value obtained for the 45-kDa GLUT1 in each fraction was multiplied by the dilution factor of each, then the PM value was divided by the total of all fractions.

### ELISA

After a 6-h fast, tail-vein blood was collected at 0 and 30 min relative to an intraperitoneal injection of 2 g/kg glucose (as per GTT). Blood samples were allowed to coagulate and then centrifuged at 17,000 × *g* for 15 min at 4°C. Serum samples were stored at −80°C. Insulin ELISA was performed using an Ultra-Sensitive Mouse Insulin ELISA kit (Crystal Chem) according to manufacturer’s instructions.

For cerebral Aβ ELISA, pulverized brain samples were weighed before extraction. Brain tissues were homogenized in 15 times their volume of ice-cold extraction buffer [Tris-buffered saline (TBS) containing 1 mm Na_3_VO_4_, 1 mm NaF, and 5 mg/ml protease inhibitors (Pierce Mini Tablets, EDTA-free)] and centrifuged at 100,000 × *g* for 60 min at 4°C. The supernatants were stored for determination of water-soluble Aβ. The pellets were washed in TBS and suspended in 50 μl extraction buffer with 1% Triton X-100 (“Deterg.” in Table 3). The suspensions were mixed using a gentle rotator for 30 min 4°C and centrifuged at 100,000 × *g* for 60 min at 4°C. These supernatants were stored for determination of detergent-soluble Aβ. The pellets were washed in TBS and resuspended in extraction buffer containing 70% formic acid (“F.A.” in Table 3). The samples were mixed on a rotator for 2 h at room temperature. After mixing, the suspensions were centrifuged at 100,000 × *g* for 60 min at 4°C. These supernatants were stored for acid-soluble Aβ analysis. All supernatants were stored at −80°C before ELISA. Aβ ELISA was performed using a human amyloid β (aa1-42) immunoassay Quantikine ELISA kit (R&D Systems).

### Primary culture of astrocytes

Astrocytes were isolated from postnatal-day-1 to -3 Sprague Dawley rat pups or mouse pups of wild-type, ApoE3-TR, and ApoE4-TR genotypes and mixed sexes. Pups were anesthetized by hypothermia, sanitized with 70% ethanol, and transferred into HEPES-buffered HBSS (Millipore Sigma). Brains were removed and the cerebral hemispheres removed from midbrain and brainstem. Meninges and olfactory bulbs were removed. Cerebral hemispheres were diced with a scalpel blade and incubated in 0.1 mg/ml trypsin (Thermo Fisher Scientific) in HBSS for 8 min at room temperature. The tissues were rinsed with HBSS and exposed to 1.5 mg/ml trypsin inhibitor (Thermo Fisher Scientific) for 10 min at room temperature. After rinsing the tissue with HBSS, the tissue was triturated and plated in tissue culture flasks with minimal essential medium (Earle’s salts; MEM) containing 10% fetal bovine serum (FBS). After 10–14 d, the loosely attached microglia were dislodged by striking the flask abruptly and washing with phosphate-buffered saline (PBS). The remaining astrocytes were then trypsinized for subculturing. For subcellular fractionation, cells were plated in 150-mm plates (three per data point). Once confluent, the culture medium was replaced with DMEM containing 0.5% FBS with 5 or 0.2 mm glucose for 24 h.

### Glucose uptake assay

Aβ_1-42_ (rPeptide) was prepared by first dissolving in hexafluoroisopropanol (HFIP) at 1 mm for 3–4 h to remove any preexisting fibrils. The HFIP was removed by evaporation, and the peptide was dissolved in anhydrous dimethyl sulfoxide at 2 mm, then diluted to 150 μm in ice-cold PBS. The solution was incubated at 4°C for 24 h to allow for the formation of oligomers.

Subcultured primary astrocytes were seeded in 24-well plates and grown to confluency. Once confluent the growth medium was replaced with MEM containing 0.5% FBS with 2.5 μm Aβ_1-42_ or vehicle for 4 h before harvest. The medium was then replaced with DMEM lacking glucose, containing 10 μCi/ml [^3^H]2-deoxyglucose and 1 μCi/ml [^14^C]sucrose. After 30 min, the medium was removed and stored for scintillation counting. The cells were washed twice with ice-cold DMEM containing 25 mm glucose then lysed via a freeze-thaw cycle in 10 mm Tris-HCl (pH 7.2) containing 0.2% sodium dodecyl sulfate. One aliquot was subjected to scintillation counting and another to protein determination by BCA reaction. The ^14^C cpm/ml in medium was used to determine the volume of extraneous medium contributing to each cell lysate, and ^3^H cpm corresponding to this volume were subtracted from the total in the lysate. Finally, the remaining ^3^H cpm were normalized to the protein content in the well.

### Statistical analysis

Datasets including both genotypes, with and without the Aβ transgene, were assessed by two-way ANOVA followed by the indicated *post hoc* test. Pearson’s correlation coefficient was used to analyze correlations. Values of *p* < 0.05 were considered statistically significant.

## Results

### Aβ significantly impairs glucose tolerance in male ApoE3-TR mice

**Figure 1.**
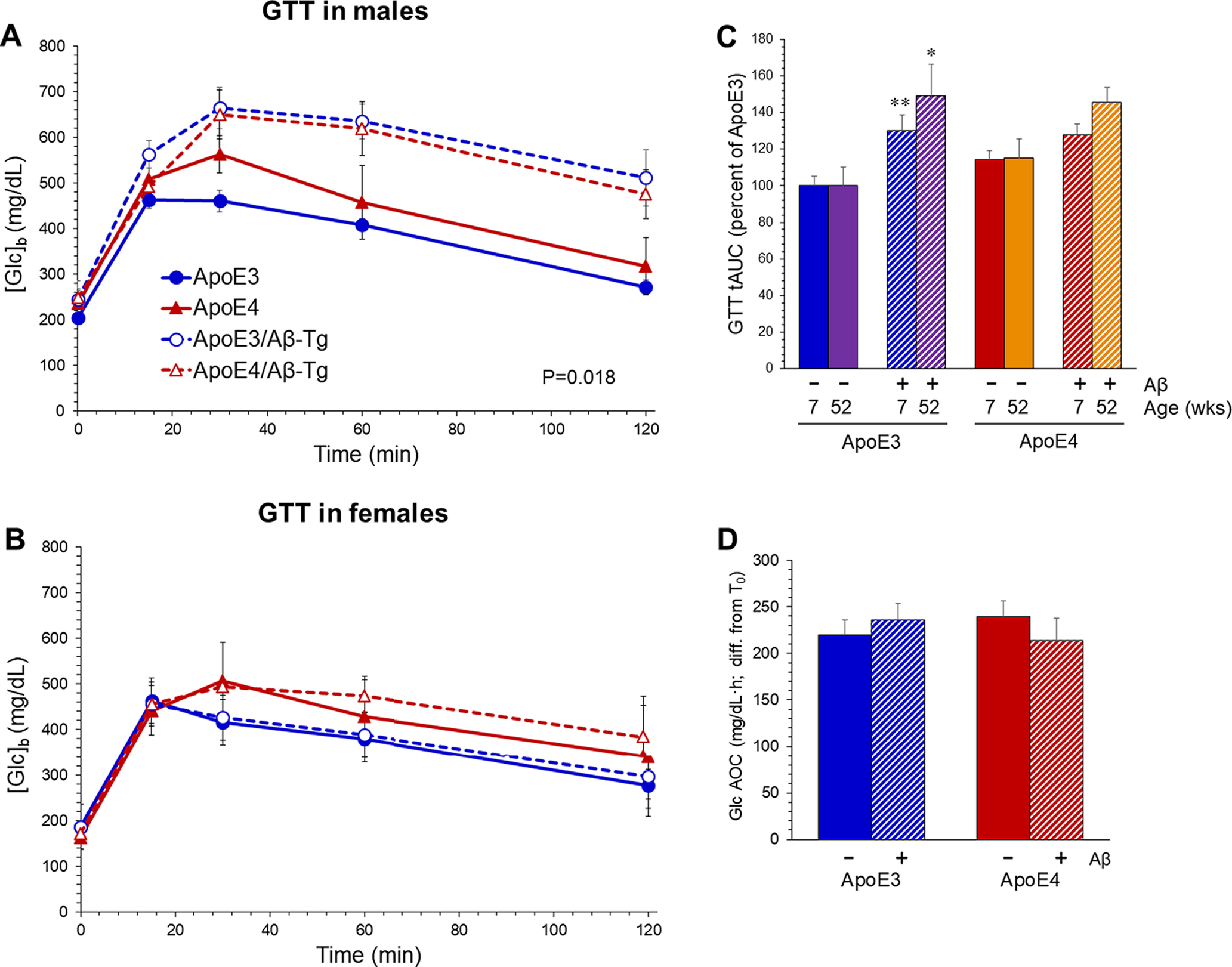
Aβ impairs glucose tolerance in male mice expressing ApoE3. Aβ-Tg mice mice were crossed with mice which had undergone targeted replacement (TR) of the endogenous *Apoe* gene with sequences producing human ApoE3 or ApoE4. They were compared with littermates which did not inherit the Aβ-Tg. GTT was performed on 12-month-old male mice (***A***) or female mice (***B***); *p* < 0.02 ApoE3-TR versus ApoE3-TR/Aβ-Tg, males only (two-way ANOVA and Bonferroni *post hoc*). ***C***, AUC was calculated from GTT values for seven-week-old and 12-month-old male mice. Results are shown relative to the mean values from ApoE3-TR mice at each age; two-way ANOVA indicated no interaction, no main effect of ApoE variant, and a main effect of Aβ (*p* < 0.01, each of the ages); *p < 0.03 versus ApoE3-TR, **p < 0.01 versus ApoE3-TR by Bonferroni *post hoc*. ***D***, ITT on males reflected as area over the curve (AOC). Values represent mean ± SEM (*N* = 7–10). In all figures, blue represents ApoE3-TR and red represents ApoE4-TR; the addition of Aβ-Tg to these ApoE genotypes is represented by cross hatching (bar graphs) or dashed line (line graphs).

We previously reported that accumulation of Aβ_1-42_ in the CNS of mice is sufficient to impair peripherally measured glucose tolerance without disruption of insulin dynamics. ApoE3-TR and ApoE4-TR mice were crossed with Aβ-Tg mice, and these were compared with their Aβ-free counterparts regarding glucose and insulin regulation. Male ApoE3-TR/Aβ-Tg mice tested at 12 months of age exhibited impairments in a glucose tolerance test (GTT) compared with ApoE3-TR mice without an Aβ transgene (Fig. 1*A*). ApoE4-TR mice trended toward an impairment, and this prevented the apparent elevation by Aβ from reaching significance. As we had observed earlier in mice with a wild-type *Apoe* locus (Hendrix et al., 2021), glucose tolerance in females expressing either of the human ApoE variants was unaffected by Aβ accumulation (Fig. 1*B*). ApoE4-TR females showed a small trend toward impairment compared with ApoE3-TR females, regardless of Aβ expression, but this did not reach significance. Body weights did not differ between the genotypes in either sex (Table 1). To test the effects of age, additional GTTs were performed on seven-week-old males. Compared with older males, the total area under the curve (tAUC) during the 2-h GTT was 29% lower in the younger ApoE3-TR and 4TR mice (without the Aβ transgene). Therefore, the results are presented as the percentage of ApoE3-TR values in each respective age group so that relative differences between the genotypes could be more readily compared across the ages (Fig. 1*C*). The relationships between ApoE variant, Aβ expression, and GTT followed essentially the same pattern in each age group, though Aβ trended toward a greater effect regardless of ApoE variant. The younger male cohort was used for the data presented henceforth.

**Table 1:** Body weights

Sex	Genotype	Body weight
Males	ApoE3	43.25 ± 2.070
	ApoE4	46.80 ± 2.881
	ApoE3/Aβ-Tg	46.47 ± 0.592
	ApoE4/Aβ-Tg	44.12 ± 0.615
Females	ApoE3	39.06 ± 4.502
	ApoE4	39.32 ± 3.444
	ApoE3/Aβ-Tg	34.07 ± 4.823
	ApoE4/Aβ-Tg	36.47 ± 3.775

Twelve-month-old mice; non-significant.

**Figure 2.**
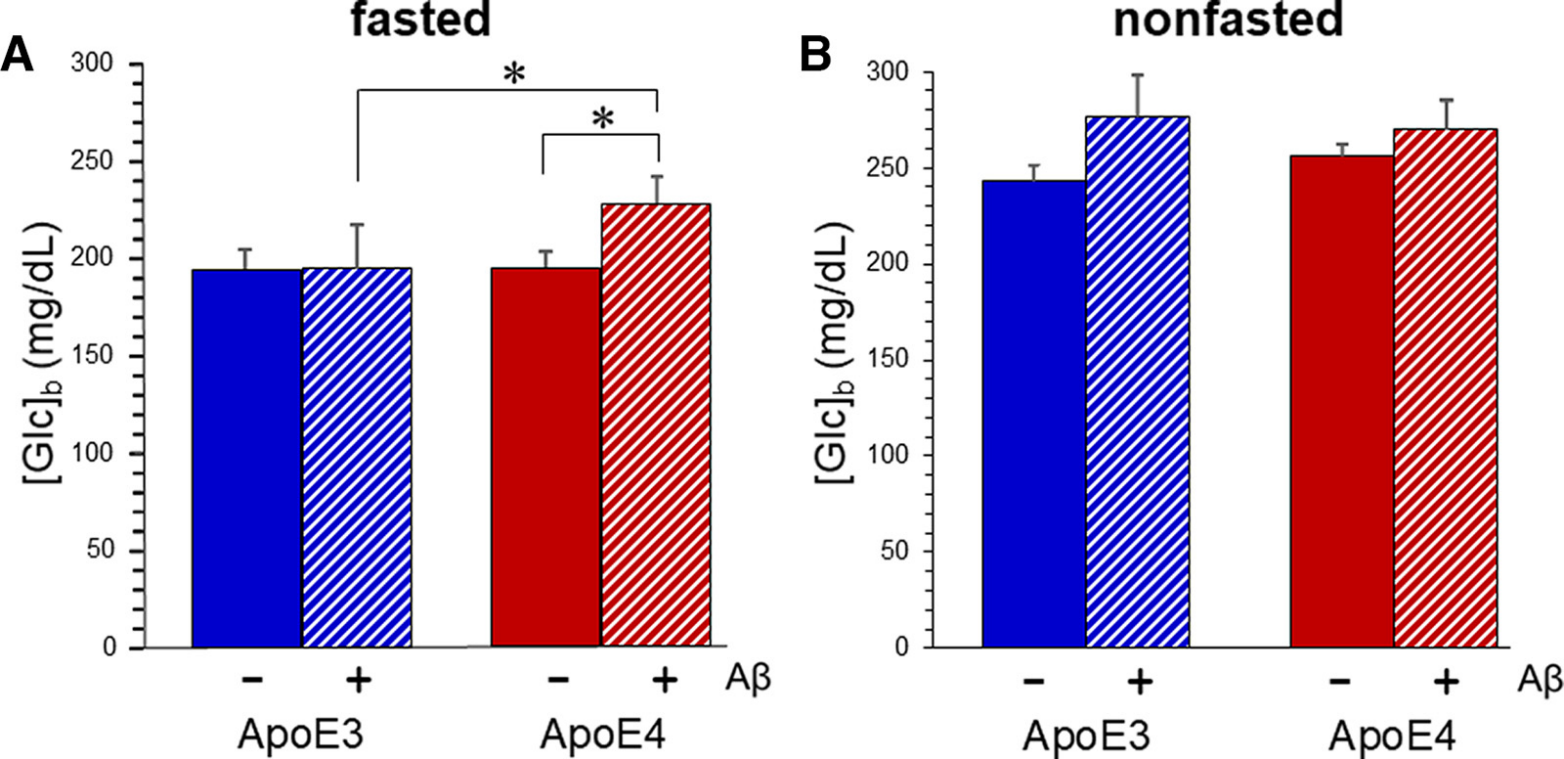
Aβ combines with ApoE4 expression to elevate basal [Glc]_b_. Basal [Glc]_b_ was measured in males of ApoE3-TR, ApoE3-TR/Aβ, ApoE4-TR, and ApoE4-TR/Aβ genotypes. The mice had either been fasted 5 h (***A***) or not (***B***). Values represent mean ± SEM (*N* = 7–10). In the fasted condition, two-way ANOVA indicated no interaction, no main effect of Aβ, and a main effect of ApoE variant (*p* < 0.05); *p < 0.03 by Bonferroni *post hoc*.

As seen in mice with a wild-type *Apoe* locus (Hendrix et al., 2021), the impaired glucose tolerance in ApoE3-TR males was not associated with insulin resistance. ITT showed no differences between any of the genotypes, as reflected in area over the curve in Figure 1*D*. Serum levels of insulin were measured after fasting (t(30)) and 30 min after intraperitoneal glucose administration (t(30)), and no significant differences between the genotypes were detected (Table 2). An effect of Aβ was noted in male ApoE4-TR mice in the form of a basal blood glucose concentration ([Glc]_b_) that remained elevated in the fasted state (Fig. 2*A*,*B*). In the nonfasted state, there was no difference in basal [Glc]_b_ among the groups. None of these effects appeared to be mediated by substantive differences in the accumulation of Aβ between ApoE3-TR and ApoE4-TR mice (Table 3).

**Table 2:** Serum insulin levels

Genotype	*t*_(0)_	*t*_(30)_
ApoE3	0.532 ± 0.086	1.16 ± 0.102
ApoE4	0.561 ± 0.145	1.08 ± 0.118
ApoE3/Aβ-Tg	0.530 ± 0.150	0.844 ± 0.096
ApoE4/Aβ-Tg	0.690 ± 0.121	1.13 ± 0.076

Seven-week-old males; non-significant.

**Table 3:** Cerebral aβ levels

Genotype	Water	Deterg.	F.A.
ApoE3/Aβ-Tg	2.400 ± 0.623	2.849 ± 0.423	3.583 ± 0.579
ApoE4/Aβ-Tg	2.672 ± 0.779	3.322 ± 0.236	3.716 ± 0.626

Seven-week-old males; non-significant.

### Aβ significantly impairs GLUT1 membrane localization in ApoE3-TR mice

**Figure 3.**
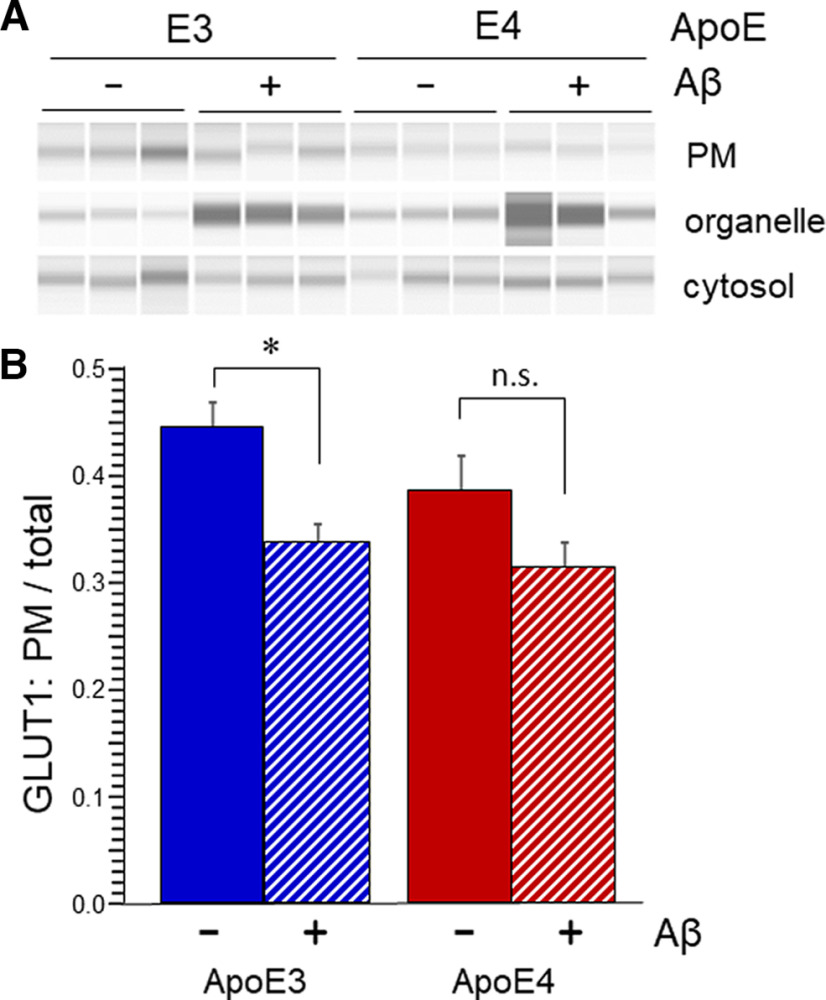
Aβ impairs plasmalemmal localization of parenchymal GLUT1 in cerebral cortex of ApoE3-TR mice. Cerebral hemispheres from ApoE3-TR, ApoE3-TR/Aβ, ApoE4-TR, and ApoE4-TR/Aβ genotypes were homogenized and fractionated to isolate plasma membrane (PM). Steady-state levels of the 45-kDa isoform of GLUT1 were determined in this fraction and the remainder of cellular components using SimpleWes capillary electrophoresis. ***A***, Virtual bands created by the SimpleWes software. ***B***, Quantification of mean PM fraction ± SEM (*N* = 6); two-way ANOVA indicated no interaction, no main effect of ApoE variant, and a main effect of Aβ (*p* < 0.002); *p < 0.02 by Bonferroni *post hoc*, n.s.: *p* = 0.08.

The impairments in GTT in male Aβ-Tg mice were previously associated with deficient trafficking of GLUT1 in brain parenchymal cells. Specifically, the proportion of the 45-kDa form of GLUT1 in cortical plasma membrane fractions was lower in these mice, as it was in AD (Hendrix et al., 2021). We examined this property in ApoE-TR males. The results revealed a pattern reciprocal to that of GTT: ApoE3-TR/Aβ-Tg mice exhibited a plasma membrane (PM) fraction of 45-kDa GLUT1 that was significantly lower than that in ApoE3-TR mice; ApoE4-TR mice trended toward a lower value, which obviated a significant difference between these and their ApoE4-TR/Aβ-Tg counterparts (Fig. 3). Total levels of GLUT1 in the four groups did not differ (data not shown).

### Both Aβ and ApoE4 are required to alter GLUT1 and glucose uptake in cultured astrocytes

**Figure 4.**
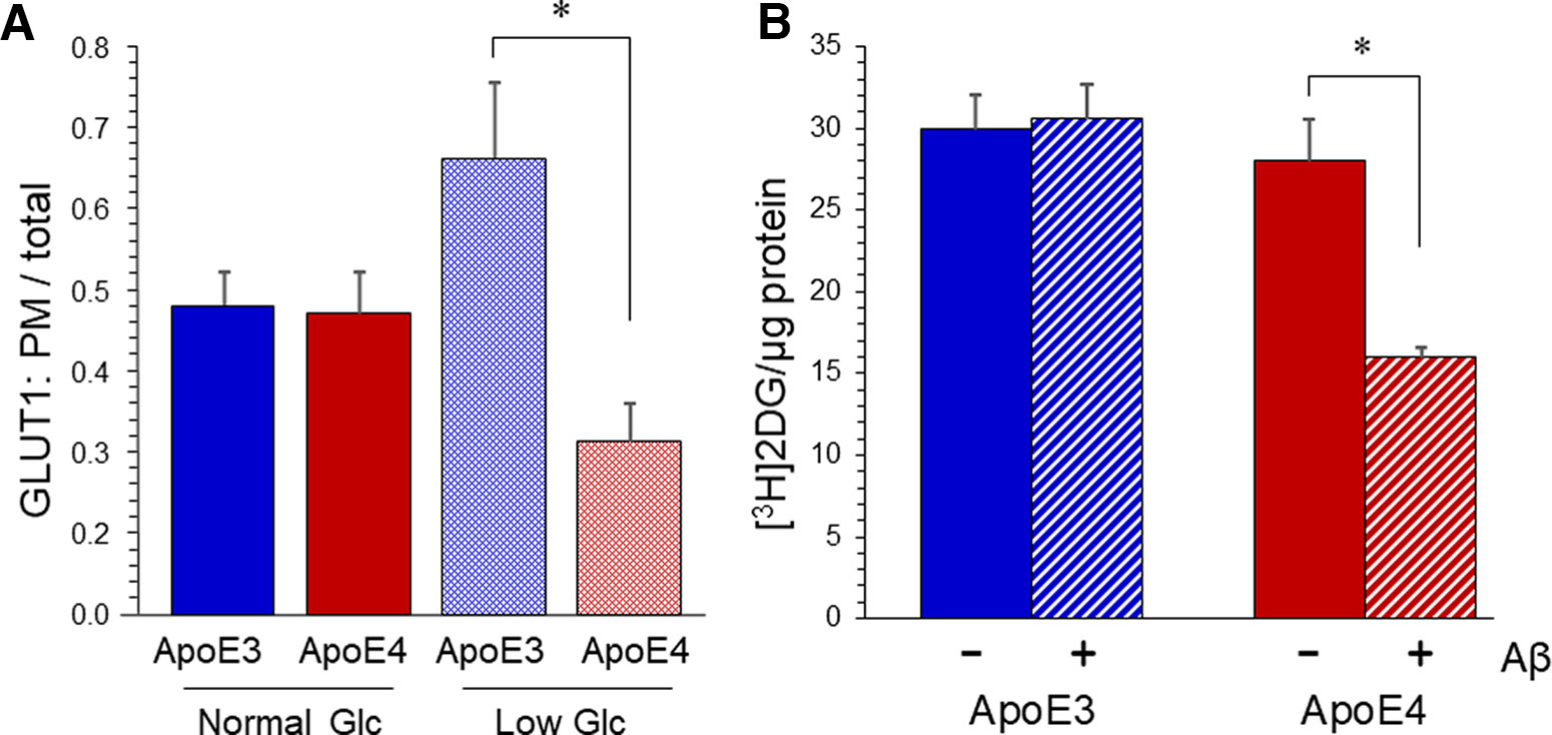
Both Aβ and ApoE4 are required to impair glucose transport in cultured astrocytes. Primary astrocyte cultures were prepared from postnatal mice of ApoE3 or ApoE4 mice. ***A***, Cells were cultured in DMEM containing 5 or 0.2 mm Glc. Homogenates were fractionated to isolate plasma membrane (PM), and levels of the 45-kDa GLUT1 were determined by SimpleWes. Quantification of mean plasmalemma fraction ± SEM (*N* = 4); two-way ANOVA indicated no main effect of [Glc], a main effect of ApoE variant (*p* < 0.02), and a significant interaction (*p* < 0.03); *p < 0.01 by Bonferroni *post hoc*. ***B***, Cultures were treated with 2.5 μm aggregated Aβ_1-42_. After 20 h, a 30-min uptake of [^3^H]2-deoxyglucose ([^3^H]2DG) was measured, corrected by inclusion of [^14^C]sucrose and normalized to protein quantity. Values represent mean ± SEM (*N* = 4); two-way ANOVA indicated a main effect of ApoE variant (*p* < 0.003), a main effect of Aβ (*p* < 0.03), and a significant interaction (*p* < 0.02); *p = 0.002 by Bonferroni *post hoc*.

We also assayed the PM fraction of 45-kDa GLUT1 in astrocytes cultured from ApoE3-TR and ApoE4-TR mice. Basal PM fraction of GLUT1 was not significantly different between ApoE3-TR and ApoE4-expressing astrocytes under normal culture conditions. As seen in prior studies with wild-type adipocytes and myocytes, reduction of glucose concentration in the culture medium enhanced the amount of GLUT1 in the PM fraction in astrocytes from ApoE3-TR mice (Fig. 4*A*). In contrast, the PM fraction of GLUT1 was deflected downward, resulting in a significant difference between the genotypes.

To test for effects of ApoE variant on glucose uptake, we assayed acute accumulation of [^3^H]2-deoxyglucose (2DG) in cultured astrocytes. Some cultures were exposed to 2.5 μm Aβ_1-42_ for 8 h before assay. (This time period was sufficient to down-regulate GLUT1 PM fraction in wild-type astrocytes.) Within a 30-min incubation with [^3^H]2DG, ApoE3-TR and ApoE4-TR astrocytes had taken up essentially equal amounts compared with ApoE4-TR astrocytes (Fig. 4*B*). Exposure to Aβ reduced the uptake of 2DG in ApoE4-TR cells, but the rate in ApoE3-TR cells was not significantly impacted by Aβ.

### ApoE4 expression elevates expression of pro-IL-1β without elevating maturation

**Figure 5.**
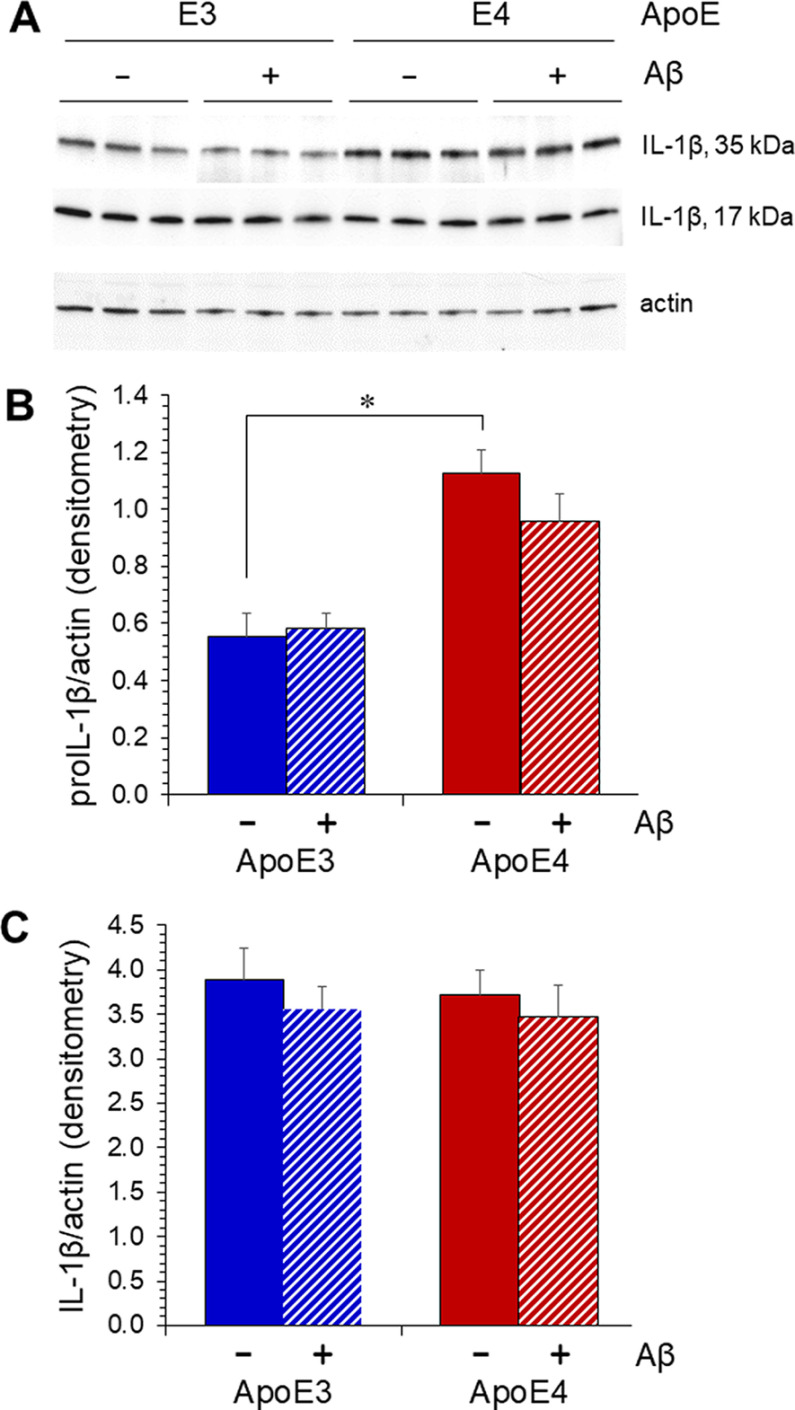
ApoE4 expression is associated with higher levels of pro-IL-1β but not mature IL-1β. Cerebral hemispheres from ApoE3-TR, ApoE3-TR/Aβ, ApoE4-TR, and ApoE4-TR/Aβ genotypes were homogenized and analyzed by Western blotting for IL-1β (***A***); β-actin was quantified as a loading control. The 35-kDa proIL-1β (***B***) and 17-kDa mature IL-1β (***C***) were quantified separately (mean ± SEM; *N* = 6). A two-way ANOVA of proIL-1β indicated no interaction, no main effect of Aβ, and a main effect of ApoE variant (*p* < 0.003); *p = 0.03 by Bonferroni *post hoc*.

Activation of inflammatory sequelae is well established in AD, and variant-specific effects of ApoE on these molecules and pathways have been reported (Vitek et al., 2009; Kloske and Wilcock, 2020). Interleukin-1β (IL-1β) is a well-documented component of the neuroinflammatory changes seen in AD and has mechanistic connections to aspects of AD pathology. In addition, we previously found that proinflammatory cytokines can impair translocation of astrocytic GLUT1 to the plasma membrane (Hendrix et al., 2021). We measured IL-1β levels in the brains of ApoE-TR males, with or without the Aβ transgene. Because maturation and release of IL-1β depends on activation of the inflammasome, we prefer to assay this cytokine by Western blotting rather than ELISA, as the former allows assessment of mobility to distinguish pro-IL-1β (35 kDa) from mature IL-1β (17 kDa). Western blot analysis of cerebral cortical tissue detected an elevation of pro-IL-1β that was dependent on ApoE4 expression and not Aβ (Fig. 5*A*,*B*). However, mature IL-1β was unaltered by either (Fig. 5*A*,*C*). The latter finding is consistent with a similar recalcitrance of interleukin-1-converting enzyme (ICE; caspase 1; data not shown).

### Tau hyperphosphorylation mirrors GTT perturbation

**Figure 6.**
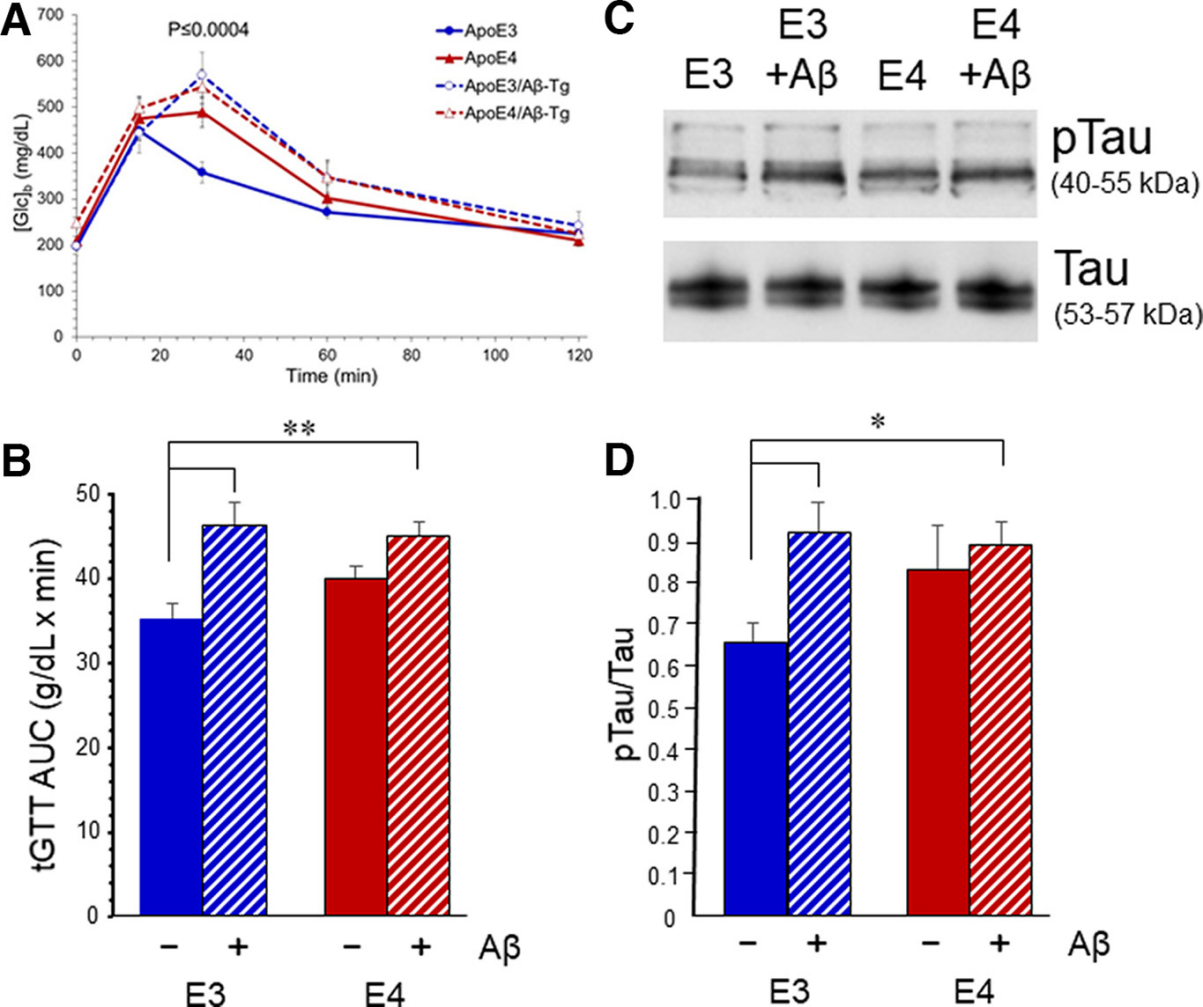
Tau pathology approximates glucose perturbation in Aβ-Tg x ApoE-TR mice. ApoE3-TR and ApoE4-TR mice were cross-bred to Aβ-Tg mice to produce males (7 weeks of age) that were homozygous for the ApoE allele and hemizygous for the Aβ transgene. ***A***, Mice were assessed in a 2-h glucose tolerance (GTT) assay. ***B***, Total AUC (mean ± SEM) for the GTT is plotted for each genotype; two-way ANOVA indicated no interaction, no main effect of ApoE variant, and a main effect of Aβ (*p* < 0.002); **p < 0.01 by Bonferroni *post hoc*. ***C***, Cerebral cortex homogenates were subjected to Western blot analysis with an antibody to phosphorylated Tau (pTau; AT8 antibody) or a phospho-independent Tau antibody. ***D***, Quantification of pTau/Tau ratio. Values are mean ± SEM; two-way ANOVA indicated no interaction, no main effect of ApoE variant, and a main effect of Aβ (*p* < 0.03); *p ≤ 0.02 by Bonferroni *post hoc*.

To determine whether markers related to neurodegeneration were altered by ApoE variant we evaluated the phosphorylation state of Tau in cerebral tissue from ApoE-TR males. Western blottings for pathologic Tau were performed with the AT8 antibody, which detects Tau phosphorylated at amino acids Ser_202_ and Thr_205_ (pTau); these sites are phosphorylated in neurofibrillary tangles found in AD and other tauopathies (Biernat et al., 1992). pTau immunoreactivity was normalized to results obtained with a phosphorylation-independent Tau antibody. GTT results were tabulated as area under the curve (AUC) to provide an integrated assessment of glucose derangement. Both parameters showed a similar step-wise increase from ApoE3-TR to ApoE4-TR to ApoE3-TR/Aβ-Tg (Fig. 6). Moreover, when the correlation was calculated between GTT AUC and pTau/Tau values for each animal, significance was observed for the overall cohort, all ApoE3-TR mice, and all mice lacking Aβ (Table 4); analysis of all ApoE4-TR and all Aβ-Tg mice showed no significance.

**Table 4:** GTT versus AT8/Tau correlation

Group	*R*	*p* value
All mice	0.46439	0.022*
All ApoE3	0.67955	0.021*
All ApoE4	−0.07666	0.824
All −Aβ	0.69847	0.013*
All +Aβ	−0.28661	0.367

*p < 0.03.

## Discussion

We previously reported that accumulation of Aβ_1-42_ in the CNS of mice is sufficient to impair glucose tolerance without disruption of the production of or sensitivity to insulin. In fact, as the utilization of glucose in the CNS is largely insulin independent, impairment of glucose tolerance in the absence of insulin resistance may be a relatively specific marker for reduced CMR_glc_. We evaluated these parameters in mice expressing human ApoE3 and ApoE4 because of the impact of *APOE* genotype on rates of AD. We expected that ApoE4 would exacerbate the physiological effects of Aβ accumulation in the CNS. To effect the latter, we used an Aβ transgene that introduces Aβ_1-42_ into the brain interstitial fluid without an APP transgene. This was deemed an important departure from prior studies because of documented effects of APP on insulin and glucose regulation. Surprisingly, perturbations of glucose tolerance did not show significant cooperative effects of Aβ and ApoE4. Instead, ApoE4 itself tended to shift values in the direction of pathophysiology. In this way, the phenotype observed in ApoE3-expressing mice showed a greater dependence on Aβ.

The results reported here are compatible with evidence that ApoE4 impacts glucose utilization independently of AD pathogenesis (e.g., in young adults; Small et al., 1995; Reiman et al., 1996, 1998). The mice used in this study were relatively young, particularly as regards plaque development in this model, which typically becomes detectable at ∼12 months of age (McGowan et al., 2005). ELISA measurement of Aβ_1-42_ did not detect significant differences in the seven-week-old ApoE3-TR versus ApoE4-TR mice. If ApoE4 significantly exacerbated Aβ accumulation in this model system, it might be expected that the combination of an Aβ transgene with ApoE4 expression would create additive or perhaps even synergistic effects, but this was not observed, even in mice that were 12 months old. A combined effect of ApoE4 and Aβ was apparent only in basal [Glc]_b_ of fasted mice. Thus, our findings suggest that ApoE4 could have detrimental effects by means other than worsening Aβ accumulation.

A few studies performed in mice combining human ApoE variants and Aβ accumulation have reported glucose dynamics, but none has directly compared ApoE-TR mice with and without the genetic manipulation of Aβ. In addition, prior studies attempted to model AD by overexpressing mutated APP. We and others have noted dramatic effects of APP on insulin and glucose regulation (Needham et al., 2008; Tu et al., 2012; Botteri et al., 2018; Kulas et al., 2019); therefore, we prefer a model of Aβ accumulation that does not depend on overexpression of APP. Pike and colleagues have made considerable study of a model (“EFAD”) wherein ApoE-TR mice are crossed with the 5xFAD line. EFAD mice responded to an obesogenic high-fat, high-sucrose (“Western”) diet in a manner reminiscent of the response we observed to Aβ: glucose tolerance was impaired by the diet-induced obesity in ApoE3-expressing mice but not in ApoE4-expressing mice. However, this relationship was shown in females (Christensen and Pike, 2019) but not males (Moser and Pike, 2017), opposite to the sex-dependency of the effects we observed. It is not clear what an increased vulnerability of females means in an animal model that does not experience menopause. While female humans develop AD at a higher rate than males, this is an entirely postmenopausal phenomenon (Seshadri et al., 1997; Riedel et al., 2016).

Several studies have evaluated glucose tolerance in ApoE-TR mice without any expression of human Aβ. Those have generally found no difference between the ApoE variants in GTT assays unless the mice were fed obesogenic diets (Arbones-Mainar et al., 2010; Jones et al., 2019; Mattar et al., 2022; Fleeman et al., 2023). Whereas female C57BL/6 mice with endogenous murine *Apoe* are generally refractory to obesogenic diets (Pettersson et al., 2012), expression of ApoE4 (but not ApoE3) seems to render them vulnerable to glucose intolerance (Jones et al., 2019). A study that exclusively used female mice found that, similar to the EFAD results described above, glucose tolerance was impaired by an obesogenic diet in ApoE3-expressing mice but not in ApoE4-expressing mice (Koren-Iton et al., 2020), whereas ApoE genotype failed to affect the response to an obesogenic diet in males (Arbones-Mainar et al., 2010). One study used mass spectrometry to assess plasma ApoE3 and ApoE4 levels in heterozygous human subjects, finding that [Glc]_b_ was inversely related to the levels of ApoE3 but not correlated with ApoE4 levels (Edlund et al., 2021). Our findings are consistent with a report of mild effects of *APOE* ε4 on GTT in human subjects (Viitanen et al., 2001).

We did not find an effect of ApoE variant on insulin resistance, consistent with results in humans (Meigs et al., 2000; Ragogna et al., 2012). Accumulating evidence suggests that metabolic disorders make a larger contribution to all-cause dementia in *APOE* ε4 noncarriers than in *APOE* ε4 carriers. For instance, *APOE* ε4 homozygotes with AD are much less likely to have hyperinsulinemia than AD patients with other genotypes (Craft et al., 1998). And ketogenic supplements, which provide a fuel source alternative to glucose, provide greater benefits to cerebral blood flow and cognition in *APOE* ε4 noncarriers (Reger et al., 2004; Henderson et al., 2009; Torosyan et al., 2018). The cognitive benefits of intranasal insulin are similarly exaggerated in *APOE* ε4 noncarriers (Reger et al., 2006). Thus, the cases of all-cause dementia among *APOE* ε4 noncarriers may include a greater number of vascular dementia, whereas *APOE* ε4 is associated with *bona fide* “pure” AD. Studies in which AD diagnosis is limited to neuropathologically confirmed cases have consistently found no association with type-2 diabetes or metabolic syndrome (Heyman et al., 1984; Hassing et al., 2002; Peila et al., 2002; Thambisetty et al., 2013; Abner et al., 2016; Groeneveld et al., 2019; Sadrolashrafi et al., 2021). Likewise, we find disparate effects of type-2 diabetes and AD on cerebral glucose transporters (our unpublished observations).

One curious finding is that basal [Glc]_b_ seemed to be modulated in a manner distinct from the conditions which impaired glucose dynamics, i.e., GTT. The latter showed a significant effect only when Aβ expressers and controls were compared in the ApoE3 background. However, this scenario did not affect basal fasted [Glc]_b_, which was altered only by the combination of ApoE4 and Aβ accumulation. Multiple mechanisms of action have been attributed to ApoE, some of which involve lipid transport or other events dependent on interaction of ApoE with its receptors. Distinct from these actions, ApoE4 can translocate to the cellular nucleus and act as a transcriptional repressor of a *cis* element termed coordinated lysosomal expression and regulation (CLEAR; Parcon et al., 2018; Lima et al., 2020). This element is otherwise bound by the transcriptional inducer TFEB to elevate expression of several genes involved in autophagy and other lysosomal processes. Thus, *APOE* ε4 may elevate the accumulation of Aβ and the risk for AD by suppressing proteostasis generally (Raha et al., 2021). The maximal GTT values reached in Aβ-Tg mice were practically identical in mice expressing ApoE3 or ApoE4. This is reminiscent of the similar binding affinities that the two variants have for lipoprotein receptors (Weisgraber et al., 1982). (ApoE2, by contrast, exhibits a much weaker interaction with LDLR and related receptors.) It is tempting to speculate that the association of ApoE variant with glucose tolerance —essentially equivalent GTT results across the two variants —is a product of receptor binding, whereas the modality in which a substantial ApoE4-dependent effect was observed —ApoE4’s elevation of basal [Glc]_b_ in Aβ-expressing mice —is associated with a bioactivity in which a biochemical difference occurs, viz. CLEAR DNA binding. Failures in proteostasis appear to play an important role in the establishment of hyperglycemia and its underlying pathophysiology (Podolsky et al., 1972; Jung et al., 2008; Ozcan et al., 2009; Bachar-Wikstrom et al., 2013).

Human studies have consistently documented lower CMR_glc_ in subjects possessing one or more *APOE* ε4 gene, even at early ages (Small et al., 1995; Reiman et al., 1996, 1998). An age-dependent reduction in glucose transport into the CNS has been reported in the ApoE-TR lines used here (Alata et al., 2015; Fang et al., 2021), though this was reported to occur at ages (8–12 months) far beyond most of the mice we examined. Surprisingly, another ApoE4-expressing model exhibits elevated CMR_glc_ as it progresses into advanced ages (Kotredes et al., 2021). ApoE4 is associated with structural and functional deficits in the cerebrovasculature (Zlokovic, 2013; Tai et al., 2016), and it is possible that this contributes to the impact of *APOE* genotype on CMR_glc_.

Effects of vascular conditions, including hyperglycemia, on CMR_glc_ typically involve downregulation of endothelial GLUT1 levels. Consistent with Alata and colleagues (Alata et al., 2015) and Wu and colleagues (Wu et al., 2018), we found no significant difference in total levels of GLUT1 (or other transporters) in ApoE4-TR mice. However, Aβ expression interfered with normal translocation of GLUT1 to the plasma membrane, as it does in mice with wild-type murine *Apoe* (Hendrix et al., 2021). This is correlated with reduced CMR_glc_ values, and we hypothesize that the insulin-independent glucose intolerance effected by Aβ represents a surrogate marker of the glucose which is not used in the CNS, due primarily to dysfunctional GLUT1. This hypothesis is supported by GTT results after conditional knock-down of GLUT1 in the astrocytes of mice (our unpublished observations). It was surprising to see that cultured astrocytes were not affected in a manner predicted by *in vivo* results. Specifically, acute treatment of ApoE3-TR astrocytes with Aβ_1-42_ did not alter [^3^H]2DG as might be expected from the results with GTT and the PM fraction of 45-kDa GLUT1 *in vivo*. Instead, it was the ApoE4-TR astrocytes that exhibited diminished glucose transport in the presence of Aβ. Several differences between *in vivo* and *in vitro* conditions may explain this discrepancy, including the acute nature of the Aβ treatment *in vitro* and the cell-type complexity of the intact cerebrum.

IL-1β is well documented as a component of neuroinflammation in AD. Previously, we found that application of inflammatory cytokines to cultured astrocytes could create the deficit in GLUT1 plasma-membrane trafficking seen in response to Aβ (Hendrix et al., 2021). Here, we found that pro-IL-1β was elevated as a function of ApoE4 expression but not possession of the Aβ transgene. Perhaps more importantly, mature IL-1β was not affected by either ApoE status or Aβ expression. One reasonable interpretation is that ApoE4 elevated transcription of the *Il1a* gene relative to ApoE3 but did not elevate activity of the inflammasome, which is responsible for proteolytic processing of pro-IL-1β (Martinon et al., 2002). Consistent with the latter, the effects of ApoE4 on inflammation have been reported to be independent of the inflammasome (Igel et al., 2021), and we detected no differences in the levels of caspase 1/interleukin-1-converting enzyme in ApoE3-TR versus ApoE4-TR mice (data not shown).

The effects of ApoE variant on Tau hyperphosphorylation mirrored those on GTT. While overall Tau levels in cerebrospinal fluid have been associated with the *APOE* ε4 genotype (Blomberg et al., 1996; Galasko et al., 1998; Tapiola et al., 1998), there is less evidence for a robust effect of ApoE variant on neurofibrillary pathology in the parenchyma (Heinonen et al., 1995; Ohm et al., 1995; Zubenko et al., 1994). Tau pathology has been correlated with aberrations in brain glucose transporters (Liu et al., 2008), and decline of CMR_glc_ is spatially correlated with higher Tau pathology detected by positon emission tomography (Ossenkoppele et al., 2016; Sun et al., 2020; Park et al., 2021), especially in the context of amyloid accumulation (Adams et al., 2019). In light of the association between impairments in GTT and CMR_glc_, our results support the idea that restricted glucose availability promotes Tau pathology, as suggested by reductionist experiments with cultured neurons (Cheng and Mattson, 1992; Lee et al., 2013).

In conclusion, we have, for the first time, compared glucose regulation in ApoE3-expressing and ApoE-expressing mice as a function of Aβ_1–42_ accumulation. We find that Aβ impairs glucose transport and tolerance in mice expressing human ApoE3 but makes less, if any, impact on these parameters in the presence of ApoE4. Thus, it is possible that Aβ accumulation is less important for development of glucose perturbation in *APOE* ε4 carriers, and—to the extent that Tau pathology mirrored these relationships—it may be less important to dementing neuropathology. It is also notable that metabolic interventions such as diet-induced obesity have been reported to be exacerbated in ApoE4-TR mice, suggesting that there are mechanistic differences in the glucose perturbations occurring in AD versus diabetes.

## Synthesis

Reviewing Editor: Aniko Korosi, Universiteit van Amsterdam Decisions are customarily a result of the Reviewing Editor and the peer reviewers coming together and discussing their recommendations until a consensus is reached. When revisions are invited, a fact-based synthesis statement explaining their decision and outlining what is needed to prepare a revision will be listed below. The following reviewer(s) agreed to reveal their identity: NONE. Note: If this manuscript was transferred from JNeurosci and a decision was made to accept the manuscript without peer review, a brief statement to this effect will instead be what is listed below.

This is a highly significant paper because it is the first time insulin signaling glucose tolerance has been evaluate in ApoE dependent. It is very well done and the discussion and results sections are very well written. There are no major concerns, but some minor concerns. The minor concerns include statistical presentation and figure presentation which are detailed below. Also the legends and the results section could be tweaked for it to be easier to interpret the findings, which make sense except require a reader slightly out of the field extra unnecessary time to make sense of it.

General Comments on Response to the review;

Strengths

• Improvement made in Response to Reviewer 1:

• Clarified issues like tau epitopes and sexes

• The authors added additional Abeta ELISA data and clarified basic question (Table 3, no changes were detected)

• Response to Reviewer 2

• Citations were added, but mostly the authors disagreed with reviewer 2, such as the problems with logic and quality of writing, but attempted to make minor accommodations to partially address Reviewer 2’s concerns, over the complexity that abeta effects on glucose are evident, but not E4 possible because both E4 and abeta impact glucose.

• Clarified issues like tau epitopes and sexes

o

• Novelty: This is the first time (surprisingly) that glucose regulation has been compared in ApoETR mice with and without Aꞵ accumulation. They also showed that glucose tolerance effects paralleled tau hyperphosphorylation and membrane translocation of astrocytic glucose transporter 1 in vitro.

Major Concerns

• None (See strengths above)

Minor Concerns and Comments

• Title :

o Suggest a slight modification to better identify the novelty; the main finding is glucose deregulation associated with E4 is sex dependent is not further enhanced by Abeta, while normal glucose regulation associated with E3 is deregulated by Abeta so for example a title like this seems more relevant. “Sex and baseline glucose degradation influence Abeta and ApoE effects on glucose deregulation"

• Methods:

were animals perfused.

•

• Reviewer Comments on graphs images were not clear; however the nature of the reviewers comments should not be a score driving issue; nevertheless below more detail on what should be changed in the graphs are made.

o Merging colors (light dark) to illustrate age or other variables for same genotype is not ideal; I suggest labeling the age or other variable on the x axis diagonally but using the same color for ApoE and always using one the colors hatched to indicate Abeta (for example ApoE3 always on color and ApoE4 always one color, but Abeta hatched and non Abeta solid.

o The legend for lines is difficult to read; I suggest slightly thicker line and use dotted or hatched lines for Abeta (analgous to above..

o Label additional information (age etc).

o Label the x axis Abeta consistently across figures graphs and Westerns. Sometimes Abeta is a row, and sometimes it is for example E3+abeta. Since some variables are added (age), it may be best to not hae Abeta as a row and have that variable as a row).

o The fonts images of the legend are often too small (e.g. for weeks). Try to make the fonts within figure as similar as possible

o

o

o Do not use the gray background in any graphs. Use white

•

Abstract

• The 1st three paragraphs are excessive introduction; please condense.

• Describe the McGowan model abeta model better the name of the line BRI-Aβ42 line and the citation, McGowan et al., 2005, and that it does not overexpress APP.

• The methods sentence(s) should add the n’s; and that astrocyte culture used af females;

• Results sentence should refer to lack of effect in females.

• Adding to overall conclusion and how it impacts the field (“this is the first time, that x,y,z”) and the lack of effect of females is surprising so I would add that (about models not mimicking menopause)

Introduction

• Last paragraph “we sought to should be a new paragraph. Delete the results; and explain the methods and variables in e.g. two sentences, and how that will answer needed questions.

Discussion

• Please revise first paragraph deleting the methods (except a preface phrase at beginning of sentences that describe results; just state the overall findings in this paragraph, and why it is important to the field. For example “despite the expectation that a,b,c would happened, because of d,e, and f, we surprisingly found that x,y, and z.”

• The sex discussion should be its own paragraph and comment on why there is a male effect at all.

• The rest of the discussion is clear and very well written.

• A short paragraph on limitations would strengthen paper eg not being able to model the sex effect.

• However, the conclusion could add a sentence of how it advance field, and what the next question is.

Figures and Legends and Statistics

• General:

o kDa’s should be shown in all images for all Westerns not just Figure 5).

o Visually the bars should be the same width if possible (fig 1,4). I suggest doing box plots or overlay data points over histogram, depending on requirements of eNEURO

o Data on females should be shown not just in figure 1; showing the negative data in females, seems extremely important to show visually as a graph, in the findings and edit legend accordingly (n=7-10, per group per sex. In which case the analysis should be two way ANOVA, and showing interaction with sex and F values of Factors (sex, transgene) I suggest adding the female as a different graph. The authors explained why these data weren’t shown; I suggest that it be shown, but it is optional; if not shown it should be repeated in Figure 2-3,5-6) that there were no impacts within females.

o The description in methods of post hoc should describe of post doc (e.g. Bonferroni or Tukey) & if there is no homogeneity of variance (required by ANOVA), if data was log transformed. It is not clear why different post-hocs were used for different graphs even within the same figure.

o One way ANOVA, seems the incorrect analysis; rather two way ANOVA, seems more appropriate. If there is a reason for one way it should be explained in methods. Otherwise, perform two way ANOVA, using factors like ApoE allele x Abeta status or x sex or x age. Please check with statistician.

o More meaningly titles should be used in text of legend. As written they are too general to appreciate main point.

Specific

Fig 1:

• Comment on legend text: need comma before which; p may need to be italicized depending on eNeuro requirements; spell out ITT and GTT (unless this is listed as an acceptable. acronym that does not require spelling out)

• Legend title, add “but not in females” or something indicating that result.

• Within the images there should be lines showing the comparisons because the comparisons for * are different , in one place asterisks is compared with E3 and the other the same single asterisk is comparison with E4. Fix Legend accordingly

• This should be two way ANOVA; it looks as if the Abeta factor is significant and the ApoE is not; and age factor non-significant; the F(df,df) = ? for these factors can be listed in legend.

• The age should not be a legend (black white) but a row in the x axis

• See above general regarding Lines.

Fig 2:

• Title: Change combines with synergizes

• The key point here is that within (fasting+ Abeta) there is an ApoE effect but no overall fasting. So since there is no effect of abeta or ApoE within non fasting, you can say that you analyzed within fasting (abeta x ApoE two way). Then say “no factors significant”. Then do a two way ANOVA for fasting (Abeta x tg) * report factors: F(df,df) =? Should be described for significant factors, which may include a Significant ApoE effect (if so report that F(df,df)=. And Factors not significant should say (ns).

• B and A should be flipped (see above)

• E4 with Abeta vs E3 is not a legitimate planned comparison and should be removed. Only put line between E4Abeta and E3Abeta.

• The y axes do not match;

• In text legend define “[Glc]b” Concentration of glucose in tail-vein blood ([Glc]b]. In image, use this nomenclature; it is used in legend but not in them image; I recommend that you not delete it form both since it is used throughout body of manuscript.

• The y axis labels should delete ‘fasted’ , ‘non fasted’ for simplicity. It is redundant to title in image (unnecessary).

Fig 3:

• Western blot image: none of the lanes are contiguous. I think it is important that some of the adjacent lanes be shown. The kDa should be listed, but they do not match likely due to the blot being curved (which is usually the case); understandably adjacent lanes are not shown because they were not loaded in that order. At least explain this in the legend (adjacent lanes are not shown because and bands to not line up because; alternatively and ideally, show adjacent lanes and label the treatment differently; for example E3 E4 E3 E4 E3/abeta E4 abeta; whatever the order.

• There is no line between ApoEs (without Abeta) but it looks as if E4 is generally impaired, so a two way ANOVA may show an ApoE effect and Abeta interaction. This could explain the interpretation better. Also is really hard to understand this figure showing plasmalemmal effect and interpretation due to the lack of explanation regarding the calculation in the text legend (and that cytosol effect was non significant?);

• Overall this is interpreted as a plasmalemmal effect but the western suggests more an organelle effect. It would be helpful to graph the organelle and cytosol separately. For example the organelle effect seems like no ApoE effect with Abeta, but E4 is higher than E3;

• Suggest deleting the word “cells” from title, since it suggest in vitro.

Fig 4:

• No Western shown for Glut1; if measure another way. Please state.

• Instead of “conspire’ I would use synergize which is more scientific, although conspire, may be okay for the body of the manuscript (but is unlikely to be acceptable to eNeuro in proofs

• The abbreviation used in the image needs to inserted into the text legend.

• The image should state “astrocytes in vitro “ or something indicating this is in vitro for ease of reading, else it blends with Figure 3 seeming redundant

• Y axis fonts are different; bar width is different (true for other figures, see general comments

• A main point of 4B, is that E4 has no problems with glucose uptake unmlesss there is Abeta; while many of the findings show pathology with E4 regardles of Abeta. This is a very important point that should be very clear in the discussion and abstract and subtitles.

• If 4B is normal glucose, put that in image (as it is already put in A). Also I suggest putting “uptake” in the image; because it is helpful and also clearer why that graph doesn’t have the low glucose group. ANOVA should be two way. It suggest and interaction between Abeta and ApoE with there be only an ApoE effect if E4 and Abeta. Since one key planned comparison should be E4+Abeta vs E3 +Abeta, especially that is the main point.

• The main point of GluT1 graphs (6A) is that

• In 4A, ApoE effect seem to goes opposite directions depending on glucose (although this may be misleading, since the significance is not shown, due to lack of homogeneity suggested by error bars, requiring a log transformation (that should be stated in the legend and the appropriate values for the log F(df,df) given or that it is ns). If it is ns, it is advisable to use the y axis log values so it doesn’t look like the low glucose effects go in opposite directions. If it is significant (response to glucose in E3, then draw the line). A two way ANOVA is likely to see a big ApoE vs glucose level interaction. Key planned comparisons E3 vs E3 low glucose vs E4 vs E4/ low glucose should be shown. This is extremely complex to interpret. A specific title should try to convey some detail. The main point for 4A is that in E3, Glut1 response to low glucose (but no line is shown to show significance) and E4 does not (or even goes in opposite direction)

• Interpretation of 4A derives a bit in part to B. so A and B should be flipped.

•

Fig. 5

• 5B: Two way ANOVA is needed; it looks like an ApoE effect, but no abeta effect and no interaction: State the that the latter F(df,df) values are non significant and the ApoE factor ( F*df,df)=?, p=?”; for proIL1, the key planned comparison is ApoE, so you should draw a parallel line over both E3s, and one over both E4s and connect, then should show one asterisk (p<0.05). The p<0.01 can be listed in the legend if necessary.

Fig. 6:

• State Age

• The main point of 6A.B is that with E4, you don’t need Abeta to show ‘pathology’; This makes a lot of sense, but is pretty hard for a reader to interpret at first glance, which is why the discussion and the subtitles should be made more clear.

• Font for 6A is too small, 6A,B are similar to Figure 1: Why isn’t 6A and 6B part of Figure 1. It is put here to compare with tau going similarly.

• 6C. suggests labeling AT8 instead pTau, then epitope and tau “total tau) you can state antibody name in legend
